# Accurate Simulation of Parametrically Excited Micromirrors via Direct Computation of the Electrostatic Stiffness

**DOI:** 10.3390/s17040779

**Published:** 2017-04-06

**Authors:** Attilio Frangi, Andrea Guerrieri, Nicoló Boni

**Affiliations:** 1 Department of Civil and Environmental Engng., Politecnico di Milano, 20133 Milano, Italy; andrea.guerrieri@polimi.it; 2 MSH Division, ST Microelectronics, 20010 Cornaredo (MI), Italy; nicolo.boni@st.com

**Keywords:** micromirrors, MOEMS, Mathieu equation, parametric resonance, continuation approach, arc length algorithm, material derivative, comb-fingers, electrostatic force and torque, electrostatic stiffness

## Abstract

Electrostatically actuated torsional micromirrors are key elements in Micro-Opto-Electro- Mechanical-Systems. When forced by means of in-plane comb-fingers, the dynamics of the main torsional response is known to be strongly non-linear and governed by parametric resonance. Here, in order to also trace unstable branches of the mirror response, we implement a simplified continuation method with arc-length control and propose an innovative technique based on Finite Elements and the concepts of material derivative in order to compute the electrostatic stiffness; i.e., the derivative of the torque with respect to the torsional angle, as required by the continuation approach.

## 1. Introduction

In the family of Micro-Opto-Electro-Mechanical-Systems (MOEMS), electrostatically-actuated torsional micromirrors are currently widely diffused in laser micro-scanners (e.g., [1]), optical shutters, micro-spectrometers, micro lenses, and pico-projectors. Indeed, electrostatic actuators can be easily fabricated using micromachining techniques, and driving voltages are compatible with standard integrated circuit (IC) technology.

Electrostatic micromirrors have been analysed in several recent papers [2,3,4,5,6,7], where it has been shown that they are governed by parametric resonance—a known phenomenon in the field of non-linear dynamics affecting, for example, the stability of ships, the forced motion of bridges, and Faraday surface wave patterns on water [8,9,10,11,12,13]. The importance of parametric amplification at the microscale has also been widely recognized for different applications (e.g., [14,15,16,17,18,19,20,21,22]).

**Figure 1:**
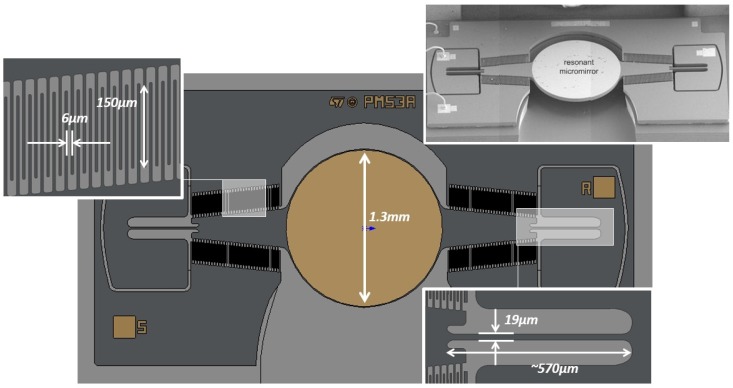
Resonant micro-mirror: SEM image of the device and layout. Courtesy of STMicroelctronics.

The 2D layout and a scanning electron microscopy (SEM) image of the silicon-on-insulator (SOI) micromirror is depicted in Figure 1. Similar components are utilized in the Intel Real Sense technology [23]. The central circular reflecting surface is attached to the substrate via two coaxial beams acting as torsional springs. Four sets of 33 fingers each are anchored to the trapezoidal regions directly attached to the mirror. These plates, interdigitated with their stator counterparts, form a comb drive structure, providing the electrostatic actuation mechanism. During operation, sensing of the opening angle is performed via the same comb drive electrodes.

Following the discussion published in [7], the mirror is treated as a rigid body hinged in its centre and connected to the substrate via the two elastically deformable springs. The torsional response is hence governed by the simple 1D model:

(1) $$\begin{array}{c} I\ddot{\psi }+B\dot{\psi }+K\psi =\varepsilon_{0}C\left(\psi \right)V^{2}\left(t\right) \end{array}$$

where $I=2.375\times 10^{10}$ ng μm${}^{2}$ is the inertia around the torsional axis, $B=1.448\times 10^{6}$ μN μm μs is a damping coefficient, $K=2.704\times 10^{7}\;\mu$N μm/rad is the torsional stiffness of the springs, and $\varepsilon_{0}C$ is the electrostatic torque due to a unit voltage bias. The voltage *V* is given in volts, and time in Equation (1) is measured in microseconds. Since $\varepsilon_{0}=8.85\times 10^{-6}$ pF/μm, *C* is measured in microns and is a purely geometrical feature. The torsional eigenfrequency of the mirror is $f_{0}=5370$ Hz. The dissipation term, in particular, is a delicate issue. Due to the presence of comb-finger actuation and of a large gap between the mirror plate and the substrate, the main contributions to dissipation are from shear flow in the comb fingers and the transport of mass induced by the large rotation of the mirror. Preliminary results have been obtained on a similar structure in [24]; however, to increase the accuracy, in the present work the constant *B* has been utilized and calibrated starting from the known maximum opening angle of the mirror in operative conditions.

It is worth stressing that, due to symmetry, the electrostatic torque around the torsional axis vanishes for ψ=0; i.e., in the rest configuration, ∀V(t). However, this trivial solution becomes unstable for some combinations of the input voltage and frequency, and triggers the mirror rotation. Indeed, in [7], it is shown that Equation (1) reduces—in the case of small rotation, negligible dissipation, and sinusoidal excitation at frequency *f*—to the well-known Mathieu’s equation [8,16]:

(2) $$\begin{array}{cc} \psi^{\prime \prime }\left(\tau \right)+\left(\delta +\epsilon \cos2\tau \right)\psi \left(\tau \right) & =0 \end{array}$$

where τ=2πft is a non-dimensional time, $\delta =f_{0}^{2}/f^{2}$, and ϵ is proportianal to the square of the maximum voltage. Equation (2) admits a nontrivial response only in specific regions of the δ,ϵ plane called instability tongues, which emanate from δ axis. In particular, the first and largest instability tongue occurs when the system is excited by a periodic forcing function at frequency $2f_{0}$, which is totally different from classical phenomena of harmonic resonance.

While the functioning of similar devices has already been extensively investigated in the literature, we focus here on a specific topic pertaining to the numerical simulation of stable and unstable branches of their response. A complete characterization of the mirror requires refined numerical techniques like the continuation approach that is briefly described in Section 2. This tool rests on an accurate evaluation of the electrostatic forcing terms and of their derivative with respect to the rotation angle. The latter term is typically computed naively with finite difference, often inducing convergence issues in the procedure. On the contrary, in Section 3 we propose a new direct procedure based on classical finite elements. Numerical results are finally discussed in Section 4.

## 2. Numerical Solution via a “Continuation” Approach

The numerical simulation of Equation (1) can be performed with several techniques. The brute force approach—which closely reproduces the actual operation of the mirror—performs a sweep over the frequencies of interest, and for each frequency simulates a sufficient number of cycles by direct integration in time to reach a steady state; the amplitude is then recorded and the next frequency is addressed, using the final amplitude and phase of the previous analysis as initial conditions. This is a very robust technique; however, it only permits the simulation of the stable branches of the amplitude vs. frequency response.

On the contrary, the continuation approach [12,25,26] with arc length control is more versatile, and is adopted herein. The model in Equation (1) is rewritten as a first-order non-autonomous differential system of equations in terms of the fictitious time τ=t/T, with τ∈[0,1]:

(3) $$\begin{array}{cc} y_{1}^{\prime } & =Ty_{2}\\ y_{2}^{\prime } & =-\frac{BT}{I}y_{2}-\frac{KT}{I}y_{1}-\frac{T}{I}\varepsilon_{0}C\left(y_{1}\right)V^{2}\left(2\pi \beta \tau \right) \end{array}$$

In Equation (3), the prime denotes differentiation w.r.t. τ, and the voltage *V* is a generic function of 2πft=2πβτ, with β=Tf. We limit our attention to periodic forcing functions with V(0)=V(2πβ) and to periodic solutions, so that our problem can be rewritten in condensed form as:

(4) $$\begin{array}{c} y^{\prime }\left(\tau \right)=TAy\left(\tau \right)+Tf\left(y_{1},\tau \right)\;\tau \in \left[0,1\right],\;y\left(0\right)=y\left(1\right) \end{array}$$

where A is a constant matrix, y collects the two unknown functions, and f contains the forcing function. It is worth stressing that, according to our assumptions: (i) In the interval *T* of analysis, f and y might contain n≥1 and m≥1 cycles, respectively, with n≠m in general. This is essential for the simulation of different instability tongues; (ii) The system could be generalized and transformed into an autonomous one by adding a nonlinear oscillator generating the desired periodic forcing, as done for instance in the software AUTO [25]. This option is not implemented herein.

**Figure 2:**
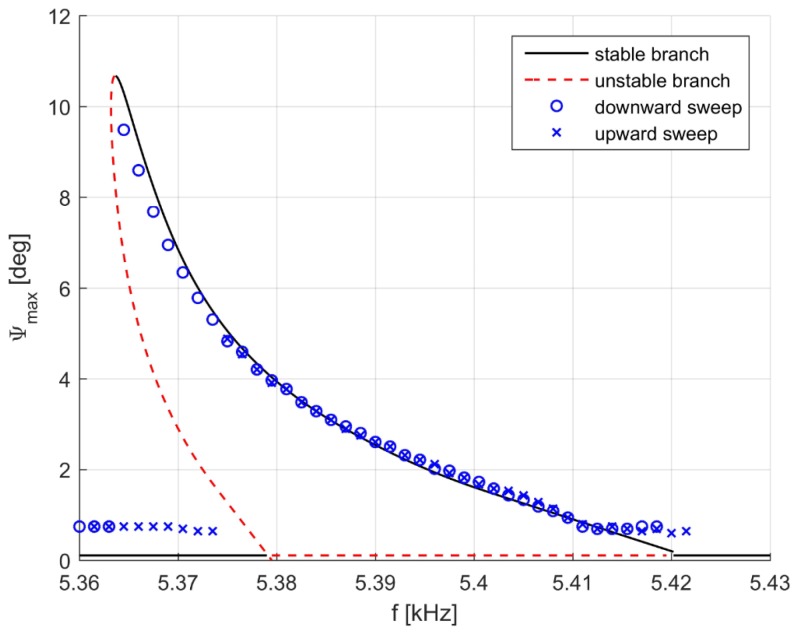
Sinusoidal excitation in the range of 5 kHz, $V_{0}$ = 55 V; experimental upward and downward sweep (discrete symbols) and numerical continuation (continuous and dashed line).

Let us suppose now that $y_{n},T_{n}$ is a known solution of the system. The simplest choice consists of taking *T* as continuation parameter: we fix $T_{n+1}=T_{n}+\Delta T$ and solve Equation (4) for $y_{n+1}$ through an iterative Newton–Raphson procedure using a suitable initial guess. However, this classical parameter continuation fails in the presence of an unstable branch as is clear from Figure 2. Indeed, by imposing an increment ΔT>0 (and hence Δf<0) at the peak, the solution would jump to the ψ=0 stable solution, completely missing the unstable dashed branches. For this reason, it is customary to introduce an arc-length control in which ΔT is part of the unknowns, the abscissa *s* along the solution branch is taken as the continuation parameter, and a new constraint F(Δy,ΔT)=0 is added. A typical choice is:

$$F\left(\Delta y,\Delta T\right)=\Delta y\cdot \Delta y+{\left(\Delta T\right)}^{2}-{\left(\Delta s\right)}^{2}=0$$

An alternative is the Keller’s pseudo arc-length method [25] in which the increment Δy,ΔT is sought such that its projection along a specific direction (typically the tangent to the y,T manifold) equals a fixed arc-length Δs.

In our simple implementation, we enforce Equation (4) in a weak manner, fixing a suitable space $C_{t}$ of vector test functions $\tilde{y}$:

(5) $$\begin{array}{cc} \mathrm{Find}\;\;y_{n+1},T_{n+1}\;\;\mathrm{such}\;\mathrm{that}\;\;y_{n+1}\left(0\right)=y_{n+1}\left(1\right)\;\;\mathrm{and}\; & \\ R\left(y_{n+1},T_{n+1};y\right)=\int_{0}^{1}\left(y_{n+1}^{\prime }-T_{n+1}Ay_{n+1}-T_{n+1}f\left(y_{1},\tau \right)\right)\;\cdot \;\tilde{y}\;d\tau & =0\;\forall \tilde{y}\in C_{t}\\ F(\Delta y,\Delta T) & =0 \end{array}$$

The segment [0–1] is partitioned into *N* equal elements, and the unknown function y is discretized over each element with quadratic Lagrangian shape functions. On the contrary, the space of test functions is selected as the space of piecewise Legendre orthogonal polynomials $P_{2}$. If Equation (5) is integrated numerically over each element with a two-point Gauss–Legendre quadrature rule, this is equivalent to the method of orthogonal collocation, in which Equation (4) is collocated at the two zeros of $P_{2}$ in each element.

Equation (5) is solved iteratively by means of a Newton–Raphson procedure. In the generic iterate, given an estimate for $y_{n+1}^{\left[k\right]},T_{n+1}^{\left[k\right]}$ (and hence for $\Delta y^{\left[k\right]},\Delta T^{\left[k\right]}$), a small correction δy,δT is sought such that

(6) $$\begin{array}{c} y_{n+1}^{\left[k+1\right]}=y_{n+1}^{\left[k\right]}+\delta y\;T_{n+1}^{\left[k+1\right]}=T_{n+1}^{\left[k\right]}+\delta T \end{array}$$

is the solution of the linearized system:

(7) $$\begin{array}{cc} \mathrm{Find}\;\;\delta y,\delta T\;\;\mathrm{such}\;\mathrm{that}\;\;\delta y(0)=\delta y(1)\;\;\mathrm{and}\; & \\ \int_{0}^{1}(\delta y^{\prime }-\delta TAy_{n+1}^{\left[k\right]}-T_{n+1}^{\left[k\right]}A\delta y-\delta Tf\left(y_{n+1}^{\left[k\right]},\tau \right)- & T_{n+1}^{\left[k\right]}\frac{\partial f}{\partial y_{1}}\left(y_{n+1}^{\left[k\right]},\tau \right)\delta y_{1})\;\cdot \;\tilde{y}\;d\tau \\ & =-R\left(y_{n+1}^{\left[k\right]},T_{n+1}^{\left[k\right]};y\right)\;\forall \tilde{y}\left(\tau \right)\in C_{t}\\ \frac{\partial F}{\partial y}\left(\Delta y^{\left[k\right]},\Delta T^{\left[k\right]}\right)\;\cdot \;\delta y+\frac{\partial F}{\partial T}\left(\Delta y^{\left[k\right]},\Delta T^{\left[k\right]}\right)\delta T & =-F(\Delta y^{\left[k\right]},\Delta T^{\left[k\right]}) \end{array}$$

The implementation of Equation (7) requires the computation of the derivative of the electrostatic torque $C(y_{1})$ with respect to the rotation angle $y_{1}=\psi$. A commonly adopted strategy is to numerically compute C(ψ) using some dedicated software and then differentiate it using standard finite differences. However, this approach often lacks the required accuracy for the iterative procedure to converge. In the following section, we detail an alternative direct formulation, based on the notion of material derivative.

## 3. Derivatives of Electrostatic Force and Torque via Direct Finite Element Computation

In this work, a finite element method (FEM) has been selected to compute the electrostatic force and torque directly by post-processing the potential field Φ of the classical electrostatic problem.

The rigid fingers of the mirror are collected in two groups: the “stator” $\Omega_{T}$ and the ”shuttle” $\Omega_{H}$—the former being fixed and the latter being movable. The stator and the shuttle are immersed in the infinite space Ω truncated at $S_{\infty }$, where homogeneous Neumann conditions are enforced. Without any loss of generality, we will assume that Φ=V on $\partial \Omega_{H}$ and Φ=0 on $\partial \Omega_{T}$, and we will express the solution as Φ=φV. The unknown function φ is governed by the following set of equations:

(8) $$\begin{array}{c} \begin{array}{cc} \nabla^{2}\varphi =0 & in\;\Omega \\ \varphi =0 & on\;\partial \Omega_{T}\\ \varphi =1 & on\;\partial \Omega_{H}\\ \nabla \varphi \cdot n=0 & on\;S_{\infty } \end{array} \end{array}$$

If $C(\bar{\varphi })$ denotes the space of sufficiently continuous functions respecting Dirichlet boundary conditions, the weak form of Equation (8) is: 

Find $\varphi \in C(\bar{\varphi })$ such that:

(9) $$\begin{array}{c} \int_{\Omega }\nabla \tilde{\varphi }\;\cdot \;\nabla \varphi \;dV=0\;\forall \tilde{\varphi }\in C\left(0\right) \end{array}$$

### 3.1. Direct Computation of Force and Torque

A straightforward way of computing forces and couples by a simple post-processing of φ flows from the notion of material derivative applied to the total potential energy [27,28,29].

Let us assume that a change of configuration Φ(x,p) is generated by the variation of a given parameter *p* (e.g., the rotation angle ψ of the shuttle), and is associated to the fictitious velocity $\theta =\left(\partial \Phi /\partial p\right)\dot{p}$. Following classical texts of optimization [27], the material derivative (also called total or particle derivative) of a scalar *f*, of a gradient ∇f and volume element dV due to θ are, respectively:

(10) $$\begin{array}{c} \overset{⋆}{f}=\frac{\partial f}{\partial p}\dot{p}+\nabla f\;\cdot \;\theta \;\overset{⋆}{\nabla f}=\nabla \overset{⋆}{f}-\nabla f\;\cdot \;\nabla \theta \;\overset{⋆}{dV}=\mathrm{div}\theta \;dV \end{array}$$

The restriction to $\partial \Omega_{H}$ of any admissible θ must be a rigid body motion U=V+ω∧x, while θ must vanish on $\partial \Omega_{T}$. The following expression for the virtual velocity will be adopted:

(11) $$\begin{array}{c} \theta \left(x\right)=\varphi \left(V+\omega \wedge x\right)=\varphi U \end{array}$$

Now:

(12) $$\begin{array}{c} \nabla \theta =U⊗\nabla \varphi +\varphi \omega^{\wedge }\;\mathrm{div}\theta =U\;\cdot \;\nabla \varphi \end{array}$$

where $\omega^{\wedge }\;\cdot \;b=\omega \;\wedge \;b$, ∀b. Let $\overset{⋆}{W}$ denote the material derivative of the total potential energy *W*. If $\varepsilon_{0}FV^{2}$ and $\varepsilon_{0}CV^{2}$ are the force and the torque exerted on $\partial \Omega_{H}$:

(13) $$\begin{array}{c} \overset{⋆}{W}={\overset{⋆}{W}}_{\mathrm{elec}}-\varepsilon_{0}F\;\cdot \;V-\varepsilon_{0}C\;\cdot \;\omega ,\;\mathrm{with}\;W_{\mathrm{elec}}=\frac{\varepsilon_{0}}{2}\int_{\Omega }\nabla \varphi \;\cdot \;\nabla \varphi \;dV \end{array}$$

where $W_{\mathrm{elec}}$ is the electrostatic energy for a unit voltage bias. Applying Equation (10):

(14) $$\begin{array}{c} {\overset{⋆}{W}}_{\mathrm{elec}}=\varepsilon_{0}\int_{\Omega }\nabla \overset{⋆}{\varphi }\;\cdot \;\nabla \varphi -\nabla \varphi \;\cdot \;\nabla \theta \;\cdot \;\nabla \varphi +\frac{1}{2}\nabla \varphi \;\cdot \;\nabla \varphi \;\mathrm{div}\theta \;dV \end{array}$$

However, the first term vanishes, since:

(15) $$\begin{array}{c} \int_{\Omega }\nabla \overset{⋆}{\varphi }\;\cdot \;\nabla \varphi \;dV=0 \end{array}$$

due to Equation (9) written with $\tilde{\varphi }\;=\;\overset{⋆}{\varphi }$. Indeed, $\overset{⋆}{\varphi }$ is an admissible test function that vanishes on both $\partial \Omega_{H}$ and $\partial \Omega_{T}$. At equilibrium, $\overset{⋆}{W}=0$, and hence:

(16) $$\begin{array}{c} F\;\cdot \;V+C\;\cdot \;\omega =\int_{\Omega }-\nabla \varphi \;\cdot \;\nabla \theta \;\cdot \;\nabla \varphi +\frac{1}{2}\nabla \varphi \;\cdot \;\nabla \varphi \;\mathrm{div}\theta \;dV \end{array}$$

yielding the final general equation:

(17) $$\begin{array}{c} F\;\cdot \;V+C\;\cdot \;\omega =-\frac{1}{2}\int_{\Omega }{\left|\;|\nabla \varphi |\;\right|}^{2}\left(\nabla \varphi \;\cdot \;U\right)\;dV \end{array}$$

since $\nabla \varphi \;\cdot \;\omega^{\wedge }\;\cdot \;\nabla \varphi =0$. In particular, the force is obtained setting ω=0

(18) $$\begin{array}{c} F=-\frac{1}{2}\int_{\Omega }{\left|\;|\nabla \varphi |\;\right|}^{2}\nabla \varphi \;dV \end{array}$$

and the torque setting V=0

(19) $$\begin{array}{c} C=-\frac{1}{2}\int_{\Omega }{\left|\;|\nabla \varphi |\;\right|}^{2}a\;dV \end{array}$$

where a=x∧∇φ. It is worth stressing that Equations (18) and (19) for the force and the torque do not involve the material derivative $\overset{⋆}{\varphi }$ of the potential. However, this is required in order to differentiate F and C.

### 3.2. Material Derivative of the Potential

Since the material derivative of the weak form Equation (9) vanishes identically:

(20) $$\begin{array}{c} \int_{\Omega }-\nabla \tilde{\varphi }\;\cdot \;\nabla \theta \;\cdot \;\nabla \varphi +\nabla \tilde{\varphi }\;\cdot \;\left(\nabla \overset{⋆}{\varphi }-\nabla \varphi \;\cdot \;\nabla \theta \right)+\nabla \tilde{\varphi }\;\cdot \;\nabla \varphi \;\mathrm{div}\theta \;dV=0 \end{array}$$

we have:

(21) $$\begin{array}{cc} \int_{\Omega } & \nabla \tilde{\varphi }\;\cdot \;\nabla \overset{⋆}{\varphi }\;dV=\int_{\Omega }{\left|\;|\nabla \varphi |\;\right|}^{2}\left(\nabla \tilde{\varphi }\;\cdot \;U\right)\;dV\\ & =\left(\int_{\Omega }{\left|\;|\nabla \varphi |\;\right|}^{2}\nabla \tilde{\varphi }\;dV\right)\;\cdot \;V+\left(\int_{\Omega }\left(x\;\wedge \;\nabla \tilde{\varphi }\right){\left|\;|\nabla \varphi |\;\right|}^{2}\;dV\right)\;\cdot \;\omega \end{array}$$

It is worth stressing that the discretization of Equation (21) leads to the same matrix as the primal problem, which can be efficiently factorized only once and solved for different rhs vectors.

Let ${\overset{⋆}{\varphi }}_{V}$ and ${\overset{⋆}{\varphi }}_{\omega }$ denote two vectors such that:

(22) $$\begin{array}{c} \overset{⋆}{\varphi }={\overset{⋆}{\varphi }}_{V}\;\cdot \;V+{\overset{⋆}{\varphi }}_{\omega }\;\cdot \;\omega \end{array}$$

The components of ${\overset{⋆}{\varphi }}_{V}$ and ${\overset{⋆}{\varphi }}_{\omega }$ can be computed from Equation (21), fixing six independent choices for U and ω.

### 3.3. Differentiation of Force and Torque

The procedure can be iterated by applying material derivatives to Equations (18) and (19). At this level, $\overset{⋆}{\varphi }$ is required. It turns out that $\overset{⋆}{F}={\overset{⋆}{F}}_{V}\;\cdot \;V+{\overset{⋆}{F}}_{\omega }\;\cdot \;\omega$, with:

(23) $$\begin{array}{cc} {\overset{⋆}{F}}_{V}=\int_{\Omega } & -\nabla \varphi \;⊗\;\left(\nabla {\overset{⋆}{\varphi }}_{V}\;\cdot \;\nabla \varphi \right)-\frac{1}{2}{\left|\;|\nabla \varphi |\;\right|}^{2}\nabla {\overset{⋆}{\varphi }}_{V}+\nabla \varphi \;⊗{\;\nabla \varphi \;|\;|\nabla \varphi |\;|}^{2}\;dV \end{array}$$

(24) $$\begin{array}{cc} {\overset{⋆}{F}}_{\omega }=\int_{\Omega } & -\nabla \varphi \;⊗\;\left(\nabla {\overset{⋆}{\varphi }}_{\omega }\;\cdot \;\nabla \varphi \right)-\frac{1}{2}{\left|\;|\nabla \varphi |\;\right|}^{2}\nabla {\overset{⋆}{\varphi }}_{\omega }+\nabla \varphi \;⊗\;\left(x\;\wedge \;\nabla \varphi \right){\left|\;|\nabla \varphi |\;\right|}^{2}\\ & +\frac{1}{2}{\varphi |\;|\nabla \varphi |\;|}^{2}\nabla \varphi^{\;\wedge \;}\;dV \end{array}$$

Similarly, $\overset{⋆}{C}={\overset{⋆}{C}}_{V}\;\cdot \;V+{\overset{⋆}{C}}_{\omega }\;\cdot \;\omega$, with:

(25) $$\begin{array}{cc} {\overset{⋆}{C}}_{V}= & \int_{\Omega }-a\;⊗\;\left(\nabla {\overset{⋆}{\varphi }}_{V}\;\cdot \;\nabla \varphi \right)-\frac{1}{2}{\left|\;|\nabla \varphi |\;\right|}^{2}x^{\;\wedge \;}\;\cdot \;\nabla^{T}{\overset{⋆}{\varphi }}_{V}+{\left|\;|\nabla \varphi |\;\right|}^{2}a\;⊗\;\nabla \varphi \\ & +\frac{1}{2}{\left|\;|\nabla \varphi |\;\right|}^{2}\nabla \varphi^{\wedge }\;dV \end{array}$$

(26) $$\begin{array}{cc} {\overset{⋆}{C}}_{\omega }= & \int_{\Omega }-a\;⊗\;\left(\nabla {\overset{⋆}{\varphi }}_{\omega }\;\cdot \;\nabla \varphi \right)-\frac{1}{2}{\left|\;|\nabla \varphi |\;\right|}^{2}x^{\;\wedge \;}\;\cdot \;\nabla^{T}{\overset{⋆}{\varphi }}_{\omega }+{\left|\;|\nabla \varphi |\;\right|}^{2}a⊗a\\ & -\frac{1}{2}{\varphi |\;|\nabla \varphi |\;|}^{2}\left(x\;⊗\;\nabla \varphi -\nabla \varphi \;⊗\;x\right)\;dV \end{array}$$

## 4. Numerical Results

While Equations (23) and (26) are general and could be applied to any rigid-body movement of the structure, for the case of interest herein, only ∂C(ψ)/∂ψ is required. The virtual velocity is a rotation around the torsional axis of unit vector e, hence V=0, ω=e, and the material derivative of the potential is $\nabla {\overset{⋆}{\varphi }}_{e}=e\;\cdot \;\nabla {\overset{⋆}{\varphi }}_{\omega }$. Moreover, $\partial C\left(\psi \right)/\partial \psi ={\overset{⋆}{C}}_{\omega }\;\cdot \;e$. Eventually, setting $\overset{⋆}{a}=x\;\wedge \;\nabla {\overset{⋆}{\varphi }}_{e}$:

(27) $$\begin{array}{cc} \frac{\partial C(\psi )}{\partial \psi }= & \int_{\Omega }-\left(a\;\cdot \;e\right)\left(\nabla {\overset{⋆}{\varphi }}_{e}\;\cdot \;\nabla \varphi \right)-\frac{1}{2}{\left|\;|\nabla \varphi |\;\right|}^{2}\left(\overset{⋆}{a}\;\cdot \;e\right)+{\left|\;|\nabla \varphi |\;\right|}^{2}{\left(a\;\cdot \;e\right)}^{2}\;dV \end{array}$$

**Figure 3:**
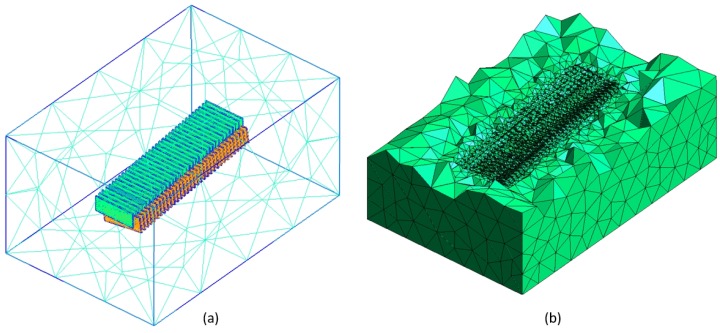
Surface mesh of one set of comb fingers (**a**) and clipping of the volume mesh (**b**).

One of the four sets of comb fingers has been simulated with a custom FEM code implementing quadratic tetrahedra, coupled with the mesh generator GMSH [30]. The surface mesh of the fingers and a clipping of the volume mesh are collected in Figure 3. The configuration considered corresponds to a rotation of ψ=0.15 rad.

**Figure 4:**
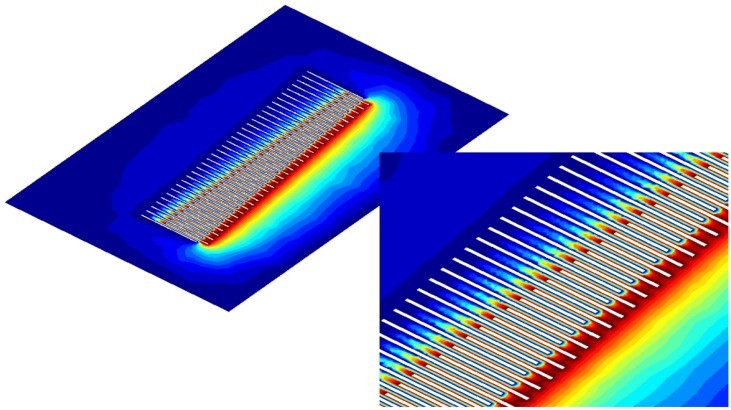
Potential φ distribution in the comb. The shuttle is set to φ=1 (red) and stator is set to φ=0 (blue).

The potential φ distribution is plotted in Figure 4. The large-scale analyses require specific hardware, but eventually compare well with the approach based on integral equations utilized in [7].

**Figure 5:**
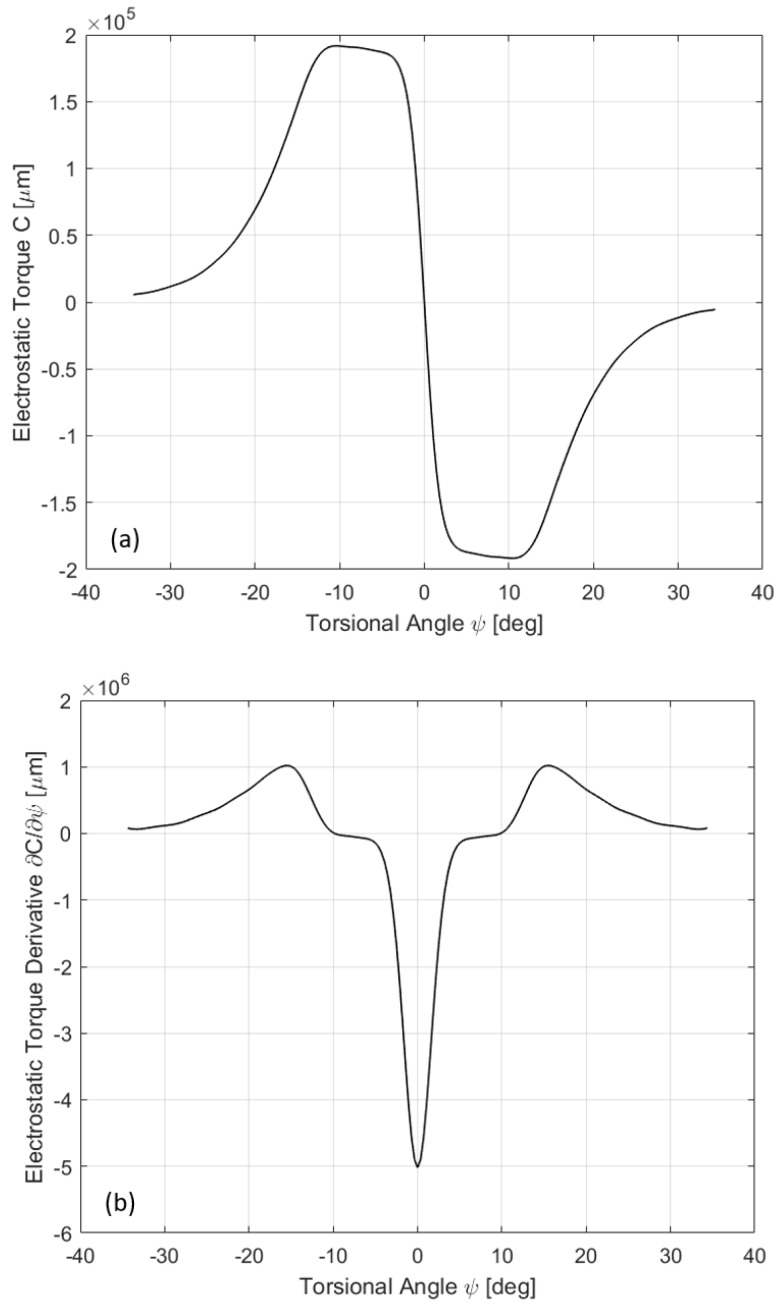
Electrostatic torque (**a**) and derivative of electrostatic torque (**b**) for one set of comb-fingers with respect the torsional angle ψ.

The computed electrostatic torque and its derivative are shown in Figure 5 for one set of comb fingers as a function of ψ. Assuming a sinusoidal excitation at frequency *f*: $V\left(t\right)=V_{0}\sin\left(2\pi ft\right)$, it is worth noting that the driving term $V^{2}\left(t\right)$ will contain the 2f harmonic. Thus, if *f* is close to the natural frequency $f_{0}$ of the mirror ($f_{0}\approx 5$ kHz), this will be excited with f≈10 kHz activating the first instability tongue. Figure 2 presents the numerical results compared with experimental data (taken from [7]), assuming a sinusoidal excitation around 5 kHz with $V_{0}=55$ V.

The ψ=0 trivial solution always exists for any *f*, but becomes unstable (red dashed line) between approximately 5380 Hz and 5420 Hz. The numerical simulation predicts the existence of two non-trivial branches: one is stable (to the right of the peak) and one is unstable (to the left of the peak). This is reflected in experimental data obtained controlling *f*, both in an upward and in a downward sweep. In the former case, *f* is increased starting from a low frequency, where ψ=0 is the only solution and is stable. However, when f≃5380 Hz the mirror becomes unstable and the system jumps to the stable non-trivial branch which is next followed up to the intersection with the trivial solution at f≃5420 Hz. Similar remarks hold when a downward sweep is performed, with the difference that the stable branch is followed until the peak is reached and next ψ suddenly drops to zero, “switching off’ the torsional movement.

The mirror response strongly depends on the voltage *V*. According to the theoretical analysis of the Mathieu equation in the presence of dissipation, a threshold $V_{0}$ exists such that below this value only the trivial response ψ=0 exists. This is confirmed by the numerical simulations: for $V_{0}=26.5$ V, the maximum aperture at the peak is 0.7 deg, and no solution is found when $V_{0}$ is further decreased.

## 5. Discussion and Conclusions

We have developed a new approach based on finite elements allowing the computation of the electrostatic force and torque exerted on solid bodies, but also the electrostatic stiffness; i.e., their derivative with respect to parameters governing the movement of the shuttle. Although the applicability of these results is quite general, our investigation has been motivated by the need to simulate phenomena of parametric resonance in electrostatically actuated micro-mirrors. The highly non-linear response of similar devices typically presents both stable and unstable branches which can be simulated using “continuation approaches”. In these techniques, starting from a known solution corresponding to a given value of a parameter, the non-linear governing equations are linearized following a Newton–Raphson procedure in order to compute the system response to a variation of the parameter. The electrostatic stiffness appears in the first-order expansion of the equation, and must be carefully evaluated in order not to spoil the quadratic convergence of the numerical scheme.

This degradation of convergence (or even lack thereof) typically arises when the torque is computed directly (or even only the capacitance), while the stiffness is obtained indirectly by applying finite difference formulas to the torque curve. This procedure does not guarantee the required accuracy. The aim of the proposed approach is to also directly compute the stiffness terms, with a precision comparable to the torque computation.

While forces and torques are based on a simple post-processing of the scalar potential of the primal problem, the computation of the electrostatic stiffness requires solving for the (material) derivative of the potential. However, this latter problem leads to the same system matrix as the primal one, thus inducing a minimal increase of computational cost.

Alternative approaches are indeed available, like those based on integral equations. However, they require dedicated codes of great complexity, while the present proposal could be easily configured as an add-on to any commercial finite element code. Clearly, a possible limitation is represented by the need of a 3D volume mesh, which leads to large-scale problems. The availability of low-cost multicore processors with large amounts of dedicated memory is, however, rapidly pushing the limit, making the solution of realistic structures on standard hardware possible.
